# Contrast-enhanced ultrasound to evaluate changes in renal cortical perfusion around cardiac surgery: a pilot study

**DOI:** 10.1186/cc12817

**Published:** 2013-07-12

**Authors:** Antoine G Schneider, Mark D Goodwin, Anthony Schelleman, Michael Bailey, Lynne Johnson, Rinaldo Bellomo

**Affiliations:** 1 Intensive Care Unit, Austin Health, Heidelberg, Victoria, Australia; 2 Radiology Department, Austin Health, Heidelberg, Victoria, Australia; 3 Australian and New Zealand Intensive Care Research Centre, School of Public Health and Preventive Medicine, Monash University, Melbourne, Victoria, Australia

**Keywords:** Renal perfusion, microcirculation, contrast-enhanced ultrasonography, cardiac surgery, peri-operative period

## Abstract

**Introduction:**

Contrast-enhanced ultrasound (CEUS) is a new technique that might enable portable and non-invasive organ perfusion quantification at the bedside. However, it has not yet been tested in critically ill patients. We sought to establish CEUS's feasibility, safety, reproducibility and potential diagnostic value in the assessment of renal cortical perfusion in the peri-operative period in cardiac surgery patients.

**Methods:**

We recruited twelve patients deemed at risk of acute kidney injury (AKI) planned for elective cardiac surgery. We performed renal CEUS with destruction-replenishment sequences before the operation, on ICU arrival and the day following the admission. Enhancement was obtained with Sonovue^® ^(Bracco, Milano, Italy) at an infusion rate of 1 ml/min. We collected hemodynamic parameters before, during and after contrast agent infusion. At each study time, we obtained five video sequences, which were analysed using dedicated software by two independent radiologists blinded to patient and time. The main output was a perfusion index (PI), corresponding to the ratio of relative blood volume (RBV) over mean transit time (mTT).

**Results:**

All 36 renal CEUS studies, including 24 in the immediate post-operative period could be performed and were well tolerated. Correlation between readers for PI was excellent (R^2 ^= 0.96, *P *< 0.0001). Compared with baseline, there was no overall difference in median PI's on ICU admission. However, the day after surgery, median PI's had decreased by 50% (*P *< 0.01) (22% decrease in RBV (*P *= 0.09); 48% increase in mTT (*P *= 0.04), both suggestive of decreased perfusion). These differences persisted after correction for haemoglobin; vasopressors use and mean arterial pressure. Four patients developed AKI in the post-operative period.

**Conclusions:**

CEUS appears feasible and well-tolerated in patients undergoing cardiac surgery even immediately after ICU admission. CEUS derived-parameters suggest a decrease in renal perfusion occurring within 24 hours of surgery.

## Introduction

Acute kidney injury (AKI) is a frequent complication of cardiac surgery and renal replacement therapy (RRT) is required in 1 to 4% of the cases [1-6]. Such severe AKI has been shown to be independently associated with increased in-hospital mortality [7].

The pathophysiology of cardiac surgery-associated AKI is still poorly understood. A decrease in renal blood flow (RBF) is believed to play a pivotal role in its pathogenesis [8,9]. There are, however, only very limited human data supporting this concept. Indeed, RBF [10] measurement, irrespective of the technique used, has only been reported in 46 critically ill patients (five studies) within the last sixty years. Thus, our knowledge, understanding, and theoretical constructs regarding renal perfusion in critically ill patients are based on extremely weak direct evidence.

Furthermore, given the complex and heterogeneous nature of the renal vasculature, some pathophysiological processes might be associated with increased global RBF [11,12] despite loss of function suggesting intra-renal shunting [13]. Therefore, techniques allowing the study of microcirculatory parameters might be more valuable in increasing our understanding of the pathophysiology of AKI. Such parameters can be evaluated by a relatively recent imaging technique, renal contrast-enhanced ultrasonography (CEUS). However, CEUS is still used infrequently in clinical practice. In particular, there are no data on the feasibility, safety, reproducibility and diagnostic value of CEUS in critically ill patients. We hypothesized that CEUS would be safe and feasible and allow quantification of changes in the renal cortical microcirculation in patients undergoing cardiac surgery.

## Methods

The study was approved by the Austin Health Research Ethics Committee (H2010/03798).

### Study protocol

We approached 12 patients planned for elective cardiac surgery and obtained informed consent. We restricted inclusion to patients deemed at high risk of AKI [14,15]. We therefore included only patients fulfilling one or more of the following criteria: age above 70 years, preexisting renal impairment (pre-operative plasma creatinine concentration >120 μmol/l), New York Heart Association (NYHA) class III/IV or impaired left ventricular function defined as left ventricular ejection fraction (LVEF) <35%, valvular surgery, redo cardiac surgery or insulin-dependent type 2 diabetes mellitus.

We excluded patients with intolerance to Sonovue^® ^(Bracco, Milano, Italy) or any other ultrasound contrast agent, end stage renal disease (plasma creatinine concentration >300 μmol/l or on haemodialysis, emergency cardiac surgery, planned off-pump cardiac surgery, known blood-borne infectious diseases, inability to obtain informed consent or enrolment in a conflicting research study. For each study patient, we performed renal CEUS on three occasions: before the surgical procedure (baseline), on ICU admission and the day after the operation.

### Ultrasound equipment and settings

We performed all measurements using an IU22^® ^ultrasound system (Philips, Amsterdam, Netherlands) with a C5-1 probe (1 to 5 MHz). We used contrast-specific mode with a low mechanical index (MI: 0.06) (R1). We set gain and depth for each patient during baseline and kept them constant for all further measurements.

We used Sonovue^® ^as the contrast agent. The agent was administered as continuous infusion at a rate of 1 ml/min using a VueJect™ syringe pump (Bracco Research, Geneva, Switzerland). After the start of the infusion, we allowed a 2-minute equilibration period and then performed and recorded five destruction-reperfusion sequences. We achieved contrast microbubble destruction by applying five pulses at high MI (flash: MI 1.24) and observed refilling at low MI (15 seconds total refilling time). We ascertained full destruction of contrast agent in the scan plane before performing destruction-reperfusion sequences.

For further examinations, to ensure that a similar portion of the renal cortex was examined, we used anatomical landmarks and visually compared the image with previously acquired sequences.

### Data analyses

We exported destruction-reperfusion sequences in digital imaging and communication in medicine (DICOM) format and analysed them using dedicated software (Sonotumor™, Bracco Research, Geneva, Switzerland). These analyses were performed by two independent radiologists (MG and TS) blinded to patient and time. In order to compensate for minor breathing artefacts, all sequences were applied with motion compensation prior to the start of the analyses.

For each sequence, one region of interest (ROI) was drawn. In order to minimize the influence of local perfusion heterogeneities, this ROI was drawn so that it enclosed all visible renal cortex on the surface of the kidney closest to the ultrasound probe. Cortex that was only intermittently visible because of breathing or other artefacts was not included in the ROI.

The software generates a perfusion index (PI), which is thought to be proportional to perfusion within an ROI. Such a PI is calculated by dividing the relative blood volume (RBV) by the mean transit time (mTT) and is expressed in arbitrary units (a.u.). These parameters have been described in detail elsewhere [16,17]. In brief, the RBV is a measure of pixel luminance and is proportional to contrast agent concentration within an ROI (it increases with higher levels of perfusion). The mTT is a measure of the time to replenishment after flash destruction of the contrast agent (it decreases with higher levels of perfusion).

For each patient and study time, the median value for the five measurements was considered for analysis. Suboptimal sequences with inadequate insonification or excessive breathing artefact were excluded as evaluated by both readers. In case of disagreement on sequence exclusion, the two readers reviewed the sequences simultaneously and consensus was reached.

### Safety parameters

For baseline studies we performed non-invasive haemodynamic (cardiac rhythm, non-invasive blood pressure and pulse oximetry) monitoring during contrast-agent infusion and for the subsequent 30 minutes. For post-operative studies full haemodynamic monitoring including invasive arterial blood pressure, pulmonary artery pressures, and cardiac index (via pulmonary artery catheter) was available for all patients.

### Statistical analysis

Analysis was performed using SAS version 9.2 (SAS Institute Inc., Cary, NC, USA). All outcomes were assessed for normality and as RBV, mTT and PI were all well-approximated by log-normal distributions, each was log-transformed prior to analysis. Inter-observer agreement was determined using correlation analyses as well as intra-class correlation [18]. For both tests, values ranged from 0 to 1 (0: absence of correlation, 1: perfect correlation). They were reported using a Bland-Altman plot with 95% limits of agreement.

Descriptive results are reported as mean (SD) for normally distributed data, otherwise as median (IQR). Longitudinal analysis determining changes from baseline was performed using mixed linear modelling with each patient treated as a random effect. Multivariable models were constructed considering all available haemodynamic and biological parameters (mean arterial pressure, cardiac index, vasoconstrictor infusion, lactate serum level, arterial pH, and serum haemoglobin level) with statistically significant variables included in the final models. A two-sided *P*-value of 0.05 was considered to be statistically significant.

## Results

### Patients' description and outcomes

Characteristics of the twelve patients are presented in Table 1. The procedure was coronary artery graft surgery (CAGS) in six patients, valvular replacement surgery (including double-valve surgery) in three and a combination of CAGS and valvular surgery in three patients. Median length of stay in hospital was 11 (8.0 to 16.5) days. All patients survived to hospital discharge.

**Table 1:** Patients' characteristics

Patient number	Body weight, Kg	Operation	CPB duration	LVEF	APACHE III score	VasoC	Diuretics	Baseline GFR, ml/min^a^	RIFLE score	Hospital length of stay, days
1	123	CABG	79	>60%	48	N	Y	142	I	8
2	101	CABG	104	>60%	36	Y	N	87	0	7
3	107	AVR + CABG	207	40%	45	Y	N	152	R	7
4	80	MVR	130	>60%	38	Y	N	98	0	32
5	76	AVR + CABG	226	20%	68	Y	Y	47	R	62
6	83	MVR + TVR	119	>60%	59	Y	Y	47	0	8
7	81	CABG	86	>60%	40	N	N	59	0	8
8	78	AVR	114	40%	32	N	N	120	0	15
9	81	CABG	69	40%	71	Y	N	59	0	15
10	78	CABG	No CPB	>60%	54	N	N	113	0	11
11	79	CABG	90	>60%	56	Y	N	52	0	11
12	106	AVR + CABG	151	>60%	74	Y	Y	86	R	18

^a^Calculated with the Cockroft-Gault equation. CPB, cardiopulmonary bypass; CABG, coronary arteries bypass graft; AVR, aortic valve replacement; MVR, mitral valve replacement; TVR, tricuspid valve replacement; LVEF, left ventricular ejection fraction; APACHE, Acute Physiology and Chronic Health Evaluation; RIFLE: risk, injury, failure, loss and end-stage renal failure; VasoC, vasoconstrictors (Y, yes; N, no); GFR, glomerular filtration rate.

### Feasibility

Although some of the scans were performed on haemodynamically unstable patients, all of them could be performed as per protocol. As an example, the scan on ICU admission was completed at a mean of 110 (SD 42) minutes after ICU admission. All scans were performed in less than half an hour (effective infusion time <8 minutes for all patients).

**Figure 1:**
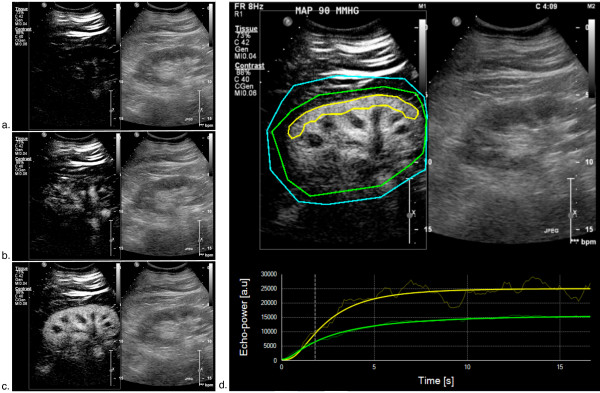
**Illustration of destruction-reperfusion sequence**. During continuous infusion of the contrast agent, microbubble destruction is obtained by applying pulses at high mechanical index (high ultrasound intensity). Microcirculation replenishment is then observed. All images represent renal contrast-enhanced ultrasonography (CEUS), the left part of the image shows contrast-image mode imaging and the right part the standard (B-mode) image. (**a**) Immediately after the flash; (**b**) during replenishment (2 seconds after the flash); (**c**) at full replenishment (6 seconds after the flash); (**d**) sequence analysis with Sonotumor^®^: a region of interest was drawn (yellow line) in the largest possible area of renal cortex closer to the ultrasound probe. The software generates a time intensity curve. This curve is used to generate CEUS-derived parameters.

Because of increased subcutaneous fluid and drains after the operation, adequate visualisation of the kidney was sometimes challenging. However, adequate contrast enhancement with contrast agent was obtained for all patients at all time points. At least one sequence for each study time point was judged by the two readers to be of adequate quality for interpretation. An illustration of destruction-refilling sequences obtained during the study is presented in Figure 1.

### Tolerance

Overall, 36 contrast-enhanced ultrasound examinations were performed using a total of 72 vials of Sonovue^® ^(10 ml per scan). Of these, twenty-four were performed in the ICU, including nine examinations in patients requiring vasoconstrictors for severe hypotension. No adverse effect was noted. Haemodynamic characteristics of patients before and after CEUS are presented in Table 2.

**Table 2:** Safety data

Measurement	Two hours pre	One hour pre	CEUS	One hour post	Two hours post	*P*-value
Cardiac index	2.87 (0.62)	2.96 (0.46)	2.9 (0.45)	2.9 (0.58)	2.92 (0.48)	0.99
Heart rate, beats per minute	84.67 (15.56)	83.83 (15.01)	84 (12.48)	84.65 (14.62)	84.88 (15.08)	0.69
Lactate, mmol/l	1.73 (1.05)	1.56 (0.84)	1.43 (0.85)	1.81 (0.87)	1.62 (1.09)	0.72
Mean arterial pressure, mmHg	80.17 (15.24)	79.96 (10.14)	78.46 (9.62)	77.17 (11.24)	76.42 (10.4)	0.43
Systolic pulmonary arterial pressure, mmHg	29.28 (15.13)	28.8 (14.17)	27.85 (14.12)	28.67 (14.74)	28.71 (15.47)	0.98
Noradrenaline infusion rate, mcg/min	1.75 (2.83)	1.88 (2.83)	1.92 (3.59)	2.5 (4.6)	2.42 (4.6)	0.94
Respiratory rate, breath per minute	15.26 (4.69)	14.96 (4.65)	16.04 (4.54)	14.83 (3.75)	15.14 (3.87)	0.39

Data are presented as mean (SD). CEUS, contrast-enhanced ultrasound.

### Inter-observer agreement

Correlation between readers was excellent for PI (*R*^2 ^= 0.96, *P *< 0.0001) and for RBV (*R*^2 ^= 0.94, *P *< 0.001) but only moderate for mTT (0.51, *P *< 0.0001). Intra-class correlation was 0.69 (95% CI 0.5, 0.84) for PI, 0.68 (95% CI 0.48, 0.83) for RBV and 0.43 (95% CI 0.25, 0.63) for mTT.

**Figure 2:**
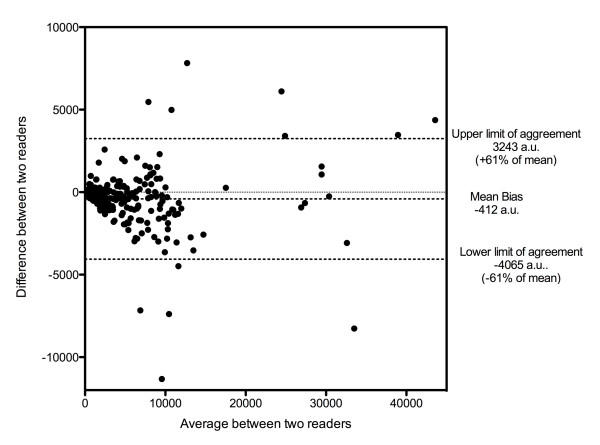
**Bland-Altman plot for inter-observer agreement (perfusion indices)**. a.u., arbitrary units.

As presented in Figure 2, agreement between readers was good. Mean bias for PI was -412 a.u. (6.9% of the mean value) with limits of agreement from -4,065 to 3,243 a.u. (± 61% of the mean value). For RBV, the mean bias was -1,320 (9.2% of the mean value) with limits of agreements from -5,670 to 3,030 (± 30.4% of the mean value). Similarly, for mTT the mean bias was -0.39 (9.1% of the mean value) with limits of agreements -7.8 to 7.1 (± 172% of the mean value).

### CEUS-derived parameters

#### Perfusion indices (PI)

**Figure 3:**
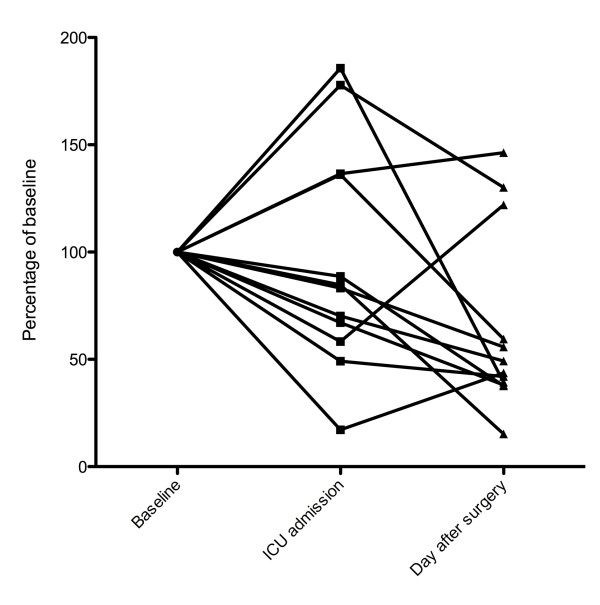
**Perfusion indices: individual patients results indexed**.

**Figure 4:**
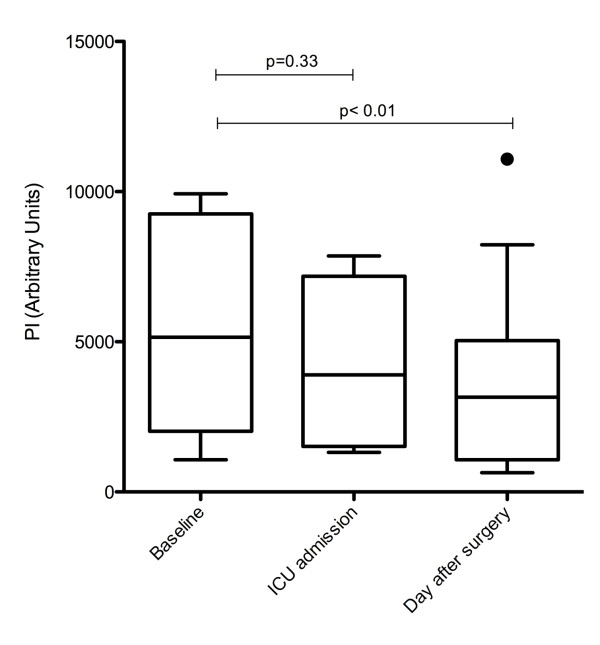
**Overall results for perfusion indices**.

Baseline PI values ranged from 1,069 to 29,446 a.u. Changes in such values indexed to baseline are presented in Figure 3. Compared with baseline, PI values decreased 24 hours after admission in nine patients. Pooled values for PI are presented in Figure 4. PI decreased from a baseline median value of 6,750 (2,042 to 8,263) to 3,936 (1,645 to 6,004) on ICU admission (-42%, *P *= 0.33) and to 3,308 (1,243 to 4,573) 24 hours later (-51%, *P *< 0.01). After adjustment for mean arterial pressure, inotrope infusion and haemoglobin, there was no difference in PI between baseline and ICU admission (*P *= 0.70) but there was a significant decrease in PI 24 hours after admission (*P *= 0.03).

#### Relative blood volume (RBV)

Baseline RBV values ranged from 4,846 to 29,958 a.u. Compared with baseline, nine patients had lower RBV 24 hours after surgery. Pooled RBV decreased from 15,342 a.u. (IQR 7,862 to 20,490) on baseline to 13,113 a.u. (IQR 8,952 to 17,310) on ICU admission (-14%, p = 0.31) and to 11929 a.u. (IQR 6,312 to 15,904) 24 hours after admission (-22%, *P *= 0.09). After adjustment for mean arterial pressure, inotrope infusion and haemoglobin, there was no difference in RBV between baseline and ICU admission (*P *= 0.29) and a trend for a decrease 24 hours after admission (*P *= 0.11).

#### Mean transit time (mTT)

Baseline mTT values ranged from 1.0 to 7.7 seconds. Compared with baseline, most (10/12) patients experienced an increase in their mTT 24 hours after the admission.

Pooled mTT increased from a median value of 2.9 seconds (IQR 2.1 to 3.3) on baseline to 3.3 (IQR 2.0 to 4.2) on ICU admission (+14%, *P *= 0.73) and to 4.3 (IQR 2.8 to 4.7) 24 hours later (+48%, *P *= 0.04). After adjustment for mean arterial pressure, inotrope infusion and haemoglobin, there was no difference in mTT between baseline and ICU admission (*P *= 0.15) or 24 hours after admission (*P *= 0.37).

### Correlation with changes in creatinine levels

Four patients developed AKI (risk, injury, failure, loss, end-stage renal failure (RIFLE)-R in three and RIFLE-I in one patient) but none required RRT. There was no correlation between changes in PI in the first 24 hours after cardiac surgery and changes in serum creatinine levels.

## Discussion

### Key findings

Using CEUS we were able to quantify changes in the microcirculation of the renal cortex before and after cardiac surgery in 12 patients deemed at risk of AKI. In these patients we performed 36 CEUS scans, including 24 in the ICU. Such studies were all performed in less than half an hour and did not interfere with clinical management. Tolerance was excellent and no adverse effect was noted. When compared with baseline, we found no overall difference in CEUS-derived parameters (PI, RBV and mTT) on ICU admission. However, 24 hours after the operation, there was an overall 50% decrease in the PI, suggestive of decreased renal cortical perfusion.

### Comparison with previous studies

The general safety of CEUS has been demonstrated in several large retrospective studies [19-21], one of which included critically ill patients [21]. The reported rates of adverse events, including potentially severe anaphylactoid reactions or complement activation-related pseudo-allergy (CARPA) [22], are in the range of 1 per 10,000 administrations. There are, however, no detailed data on CEUS safety after cardiopulmonary bypass, or during mechanical ventilation or vasoconstrictor administration. Our study provides such information. In addition, we were able to report the absence of changes in systolic pulmonary pressure after contrast-agent administration. This is consistent with previous findings [23] in patients undergoing right heart catheterization with Definity^® ^as a contrast agent. Our data confirm the absence of measurable physiological changes even in haemodynamically unstable patients during administration of Sonovue^® ^for CEUS.

In a previous study [24], we evaluated the ability of CEUS to detect changes in renal cortical perfusion in healthy volunteers. We found that CEUS was able to detect a 15% change in renal flow as induced by angiotensin II or captopril administration. These results were consistent with those found by Kishimoto *et al*. [25] using a different technique. However, to the best of our knowledge, there has not been any previous attempt to use CEUS for quantification of renal perfusion in patients undergoing cardiac surgery, hence our findings are novel and cannot be compared with previous data. Estimates of global renal blood flow have been obtained with the measurement of para-immuno hippurate clearance corrected by renal vein sampling [26]. This technique requires the insertion of an 8-Fr catheter into the left renal vein under fluoroscopic guidance. Using this invasive strategy, authors have demonstrated, in similar patients, the potential of noradrenaline to improve oxygen delivery, glomerular filtration rate (GFR) and the renal oxygen supply/demand relationship in cardiac surgery patients with vasodilatory shock and AKI [27], as well as an increase in RBF induced by mannitol [28] or dopamine [29]. The lack of correlation between estimate of flow and function is consistent with data obtained with cine-phase contrast magnetic resonance imaging (MRI) [30].

### Clinical significance

Our pilot study supports the feasibility and safety of CEUS at the bedside in the ICU, in particular in the period around cardiac surgery, even in patients deemed at risk of AKI. Despite some relative haemodynamic instability, adequate visualisation of the kidney was feasible in all cases. The finding of decreased cortical perfusion at 24 hours is plausible and consistent with previous findings [30]. This study, together with larger safety studies, establishes CEUS as a fast, safe and feasible procedure in critically ill patients. Larger studies are required to confirm or refute the decrease in renal cortical perfusion after cardiac surgery suggested by our results.

### Strength and limitations

To the best of our knowledge, this study is the first to report non-invasive, real-time measurement of renal cortical microcirculation in humans before and after cardiac surgery. In addition, it has several strengths. Extensive invasive monitoring was available for all patients in the post-operative period, enabling detection of subclinical adverse events. All scans were performed by a single operator (AS) and were analysed by two senior radiologists blinded to patient and time. This enabled evaluation of inter-observer agreement. Despite important baseline heterogeneity, a statistically significant decrease in renal cortical perfusion was detected with a sample size as small as 12 patients.

On the other hand, this study has several limitations. First, CEUS parameters could not be correlated to a comparator/gold standard. Indeed, such measurements of the microcirculation are novel and no gold standard has emerged. Comparison with macrocirculation using techniques such as PAH clearance or MRI would have been theoretically possible but logistically very complicated or invasive and could not occur simultaneously. In addition, such comparison would be informative at best, but could also potentially be misleading, as the correlation between macro- and microcirculation is not necessarily linear.

There was important heterogeneity in individual results and baseline perfusion indices. However, such heterogeneity was not likely to arise from interpretation errors, as illustrated by the good agreement between the readers. Similar heterogeneity in baseline measurements was found in our previous study in healthy subjects [24]. It is more likely to be associated with different patient properties, such as depth of organ, thickness of subcutaneous tissue and the renal capsule or other properties influencing ultrasound beam attenuation. This is consistent with the greater variability observed in the RBV component, which is more sensitive to attenuation (as it is a measure of the intensity of the ultrasound signal). The mTT component, which is the time to replenishment, could be a more robust measure. However, this heterogeneity should not have influenced the validity of our results, as only changes in values from baseline in identical patients were considered. Decreased renal perfusion was not found in all patients. However, patients had different clinical courses and heterogeneity would be expected, given the differences in age, type of operation and duration of bypass.

We did not find a correlation between CEUS indices and impairment of renal function; however, given the small number of patients, this needs to be confirmed in larger studies. Finally, we were not able to report on medullary perfusion. Indeed, such a parameter together with cortico-medullary perfusion ratios of CEUS parameters would be of great interest. However, medullary perfusion was not measurable with adequate reproducibility using our current technology.

### Future studies

Further studies are required to confirm or refute these results. In particular, due to the limited number of patients included in this study, we were not able to draw any conclusion about correlation of changes in CEUS-derived indices and clinical outcomes such as AKI, need for RRT or mortality.

## Conclusions

CEUS is feasible and well-tolerated in patients undergoing cardiac surgery, in particular immediately after ICU admission. CEUS-derived parameters suggest a decrease in renal perfusion occurring within 24 hours of surgery. Further studies with larger sample size are required to establish whether there is a correlation between changes in microvascular cortical flow and markers of renal function.

## Key messages

• CEUS is safe and feasible in critically ill patients and in particular during the cardiac surgery peri-operative period.

• In patients at risk of AKI, CEUS-derived parameters suggest a decrease in renal cortical perfusion in the 24 hours following cardiac surgery.

## Abbreviations

AKI: acute kidney injury; APACHE: Acute Physiology and Chronic Health Evaluation; AVR: aortic valve replacement; a.u.: arbritrary units; CABG: coronary arteries bypass graft; CAGS: coronary artery graft surgery; CARPA: complement activation-related pseudo-allergy; CEUS: contrast-enhanced ultrasound; CPB: cardiopulmonary bypass; GFR: glomerular filtration rate; DICOM: digital imaging and communication in medicine; LVEF: left ventricular ejection fraction; MRI: magnetic resonance imaging; MI: mechanical index; mTT: mean transit time; MVR: mitral valve replacement; NYHA: New York Heart Association; PI: perfusion index; RBF: renal blood flow; RBV: relative blood volume; RIFLE: risk, injury, failure, loss, end-stage renal failure; ROI: region of interest; RRT: renal replacement therapy; TVR: tricuspid valve replacement.

## Competing interests

The authors declare that they have no competing interests.

## Authors' contributions

AGS participated in study design, carried out participant recruitment, performed CEUS, participated in data interpretation and drafted the manuscript. MG participated in study design and performed data analyses using dedicated software. AS participated in study design and performed data analyses using dedicated software. MB participated in study design and performed statistical analyses. LJ participated in study design and contributed to CEUS. RB participated in study design and data interpretation, and critically reviewed the manuscript. All authors read and approved the final manuscript.

## References

[B1] Mariscalco G Lorusso R Dominici C Renzulli A Sala A Acute kidney injury: a relevant complication after cardiac surgery Ann Thorac Surg 2011 17 1539 1547 10.1016/j.athoracsur.2011.04.123 21872837

[B2] Conlon PJ Stafford-Smith M White WD Newman MF King S Winn MP Landolfo K Acute renal failure following cardiac surgery Nephrol Dial Transplant 1999 17 1158 1162 10.1093/ndt/14.5.1158 10344355

[B3] Andersson LG Ekroth R Bratteby LE Hallhagen S Wesslen O Acute renal failure after coronary surgery - a study of incidence and risk factors in 2009 consecutive patients Thorac Cardiovasc Surg 1993 17 237 241 10.1055/s-2007-1013861 8211928

[B4] Mangano CM Diamondstone LS Ramsay JG Aggarwal A Herskowitz A Mangano DT Renal dysfunction after myocardial revascularization: risk factors, adverse outcomes, and hospital resource utilization. The Multicenter Study of Perioperative Ischemia Research Group Ann Intern Med 1998 17 194 203 10.7326/0003-4819-128-3-199802010-00005 9454527

[B5] Thakar CV Liangos O Yared JP Nelson D Piedmonte MR Hariachar S Paganini EP ARF after open-heart surgery: Influence of gender and race Am J Kidney Dis 2003 17 742 751 10.1016/S0272-6386(03)00021-0 12666060

[B6] Ostermann ME Taube D Morgan CJ Evans TW Acute renal failure following cardiopulmonary bypass: a changing picture Intensive Care Med 2000 17 565 571 10.1007/s001340051205 10923731

[B7] Chertow GM Levy EM Hammermeister KE Grover F Daley J Independent association between acute renal failure and mortality following cardiac surgery Am J Med 1998 17 343 348 10.1016/S0002-9343(98)00058-8 9576407

[B8] Schrier RW Wang W Acute renal failure and sepsis N Engl J Med 2004 17 159 169 10.1056/NEJMra032401 15247356

[B9] Schneider A Goodwin M Bellomo R Measurement of kidney perfusion in critically ill patients Crit Care 2013 in press 10.1186/cc12529 PMC3672528 23514525

[B10] Prowle JR Ishikawa K May CN Bellomo R Renal blood flow during acute renal failure in man Blood Purif 2009 17 216 225 10.1159/000230813 19648741

[B11] Ishikawa K Calzavacca P Bellomo R Bailey M May CN Effect of selective inhibition of renal inducible nitric oxide synthase on renal blood flow and function in experimental hyperdynamic sepsis* Crit Care Med 2012 17 2368 2375 10.1097/CCM.0b013e3182514be9 22622397

[B12] Morimatsu H Ishikawa K May CN Bailey M Bellomo R The systemic and regional hemodynamic effects of phenylephrine in sheep under normal conditions and during early hyperdynamic sepsis Anesth Analg 2012 17 330 342 10.1213/ANE.0b013e31825681ab 22584559

[B13] O'Connor PM Evans RG Structural antioxidant defense mechanisms in the mammalian and nonmammalian kidney: different solutions to the same problem? Am J Physiol Regul, Integr Comp Physiol 2010 17 R723 727 10.1152/ajpregu.00364.2010 20660108

[B14] Haase M Haase-Fielitz A Bellomo R Devarajan P Story D Matalanis G Reade MC Bagshaw SM Seevanayagam N Seevanayagam S Doolan L Buxton B Dragun D Sodium bicarbonate to prevent increases in serum creatinine after cardiac surgery: a pilot double-blind, randomized controlled trial Crit Care Med 2009 17 39 47 10.1097/CCM.0b013e318193216f 19112278

[B15] Burns KE Chu MW Novick RJ Fox SA Gallo K Martin CM Stitt LW Heidenheim AP Myers ML Moist L Perioperative N-acetylcysteine to prevent renal dysfunction in high-risk patients undergoing cabg surgery: a randomized controlled trial JAMA 2005 17 342 350 10.1001/jama.294.3.342 16030279

[B16] Arditi M Frinking PJ Zhou X Rognin NG A new formalism for the quantification of tissue perfusion by the destruction-replenishment method in contrast ultrasound imaging IEEE Trans Ultrason Ferroelectr Freq Control 2006 17 1118 1129 16846144 10.1109/tuffc.2006.1642510

[B17] Schneider A Johnson L Goodwin M Schelleman A Bellomo R Bench-to-bedside review: Contrast enhanced ultrasonography - a promising technique to assess renal perfusion in the ICU Crit Care 2011 17 157 10.1186/cc10058 21586101 PMC3218962

[B18] Bland JM Altman DG Statistical methods for assessing agreement between two methods of clinical measurement Lancet 1986 17 307 310 2868172

[B19] Main ML Ryan AC Davis TE Albano MP Kusnetzky LL Hibberd M Acute mortality in hospitalized patients undergoing echocardiography with and without an ultrasound contrast agent (multicenter registry results in 4,300,966 consecutive patients) Am J Cardiol 2008 17 1742 1746 10.1016/j.amjcard.2008.08.019 19064035

[B20] Kusnetzky LL Khalid A Khumri TM Moe TG Jones PG Main ML Acute mortality in hospitalized patients undergoing echocardiography with and without an ultrasound contrast agent: results in 18,671 consecutive studies J Am Coll Cardiol 2008 17 1704 1706 10.1016/j.jacc.2008.03.006 18436124

[B21] Wei K Mulvagh SL Carson L Davidoff R Gabriel R Grimm RA Wilson S Fane L Herzog CA Zoghbi WA Taylor R Farrar M Chaudhry FA Porter TR Irani W Lang RM The safety of deFinity and Optison for ultrasound image enhancement: a retrospective analysis of 78,383 administered contrast doses J Am Soc Echocardiogr 2008 17 1202 1206 10.1016/j.echo.2008.07.019 18848430

[B22] Szebeni J Complement activation-related pseudoallergy: a new class of drug-induced acute immune toxicity Toxicology 2005 17 106 121 10.1016/j.tox.2005.07.023 16140450

[B23] Wei K Main ML Lang RM Klein A Angeli S Panetta C Mikati I Lee LV Bernstein JA Ahmad M The effect of Definity on systemic and pulmonary hemodynamics in patients J Am Soc Echocardiogr 2012 17 584 588 10.1016/j.echo.2012.01.019 22365709

[B24] Schneider AG Hofmann L Wuerzner G Glatz N Maillard M Meuwly JY Eggimann P Burnier M Vogt B Renal perfusion evaluation with contrast-enhanced ultrasonography Nephrol Dial Transplant 2012 17 674 681 10.1093/ndt/gfr345 21690200

[B25] Kishimoto N Mori Y Nishiue T Shibasaki Y Iba O Nose A Uchiyama-Tanaka Y Masaki H Matsubara H Iwasaka T Renal blood flow measurement with contrast-enhanced harmonic ultrasonography: evaluation of dopamine-induced changes in renal cortical perfusion in humans Clin Nephrol 2003 17 423 428 10.5414/CNP59423 12834173

[B26] Sward K Valsson F Sellgren J Ricksten SE Bedside estimation of absolute renal blood flow and glomerular filtration rate in the intensive care unit. A validation of two independent methods Intensive Care Med 2004 17 1776 1782 15375650 10.1007/s00134-004-2380-8

[B27] Redfors B Bragadottir G Sellgren J Sward K Ricksten SE Effects of norepinephrine on renal perfusion, filtration and oxygenation in vasodilatory shock and acute kidney injury Intensive Care Med 2011 17 60 67 10.1007/s00134-010-2057-4 20949349

[B28] Bragadottir G Redfors B Ricksten SE Mannitol increases renal blood flow and maintains filtration fraction and oxygenation in postoperative acute kidney injury: a prospective interventional study Crit Care 2012 17 R159 10.1186/cc11480 22901953 PMC3580749

[B29] Redfors B Bragadottir G Sellgren J Sward K Ricksten SE Dopamine increases renal oxygenation: a clinical study in post-cardiac surgery patients Acta Anaesthesiol Scand 2010 17 183 190 10.1111/j.1399-6576.2009.02121.x 19764906

[B30] Prowle JR Molan MP Hornsey E Bellomo R Measurement of renal blood flow by phase-contrast magnetic resonance imaging during septic acute kidney injury: a pilot investigation Crit Care Med 2012 17 1768 1776 10.1097/CCM.0b013e318246bd85 22487999

